# Child Safety First: A Public Health Initiative to Address Pediatric Non-Accidental Burns in Central Europe

**DOI:** 10.3389/ijph.2025.1608291

**Published:** 2025-08-06

**Authors:** Julia Bartkova, Rei Ogawa

**Affiliations:** ^1^ Department of Burns and Plastic Surgery, Faculty of Medicine, Institution Shared with University Hospital Brno, Masaryk University, Brno, Czechia; ^2^ Department of Plastic, Reconstructive and Aesthetic Surgery, Nippon Medical School, Tokyo, Japan

**Keywords:** burns, children, NAB, pediatric burns, non-accidental burns

**The IJPH series “Young Researcher Editorial” is a training project of the Swiss School of Public Health.**

Non-accidental burns (NAB) refer to burn injuries in children that were not caused by accident, but were intentionally inflicted, often as a result of abuse or neglect. Healthcare providers caring for pediatric burn patients must assess whether the injuries were accidental or deliberate. If the burns are determined to be non-accidental, appropriate steps must be taken to ensure the child’s safety. According to the American Burn Association, over 300 children are treated in emergency departments for burn injuries each day, and approximately two children die daily as a result. Children under 16 years of age represent about 26% of all admissions to burn centers [1]. International estimates of the incidence of NAB in children has been shown to vary from as low as 1.7% [2] to up to 25% [3] of burns unit admissions. Data from burn units in the UK show an incidence of 3% [4]. Figures from burn units in the USA show an incidence of 24% [5]. There is little NAB data for Central Europe because reporting systems are not standardized across the region and data collection is inconsistent. Systematic data collection is difficult because counties have different healthcare infrastructure. Cultural factors also play a role, e.g., willingness to report abuse and obligations to protect data. Here, we argue for a unified, evidence-based screening framework that protects children from NAB, ensures equitable care, and improves public health outcomes.

Children with NAB are often severely injured and sometime killed [6]. Treating burns is resource intensive; burned children may need skin grafts, spend a long time in the hospital, and require extensive outpatient follow-up [7]. NAB is harder to diagnose than many other forms of child abuse [6] since burns may appear accidental, and children may be reluctant to explain how they occurred. NAB patterns vary, and when there is no clear evidence of abuse it takes careful investigation to distinguish between accidental injuries and NAB.

Even though central Europe’s healthcare infrastructure supports specialized burn centers that can manage complex burn injuries and burn patients in the region are typically transported to dedicated burn centers where they can access specialized care and advanced treatment options, there is no standardized protocol to guide healthcare workers in cases of suspected NAB. Healthcare providers often make subjective judgments, report inconsistently, and may be prone to underdiagnose NAB. The socioeconomic disparities and stigma associated with abusive NAB may impede timely intervention. NAB caused by abuse may also be misclassified as accidental when healthcare professional lack training or awareness [8, 9]. Without national databases to track child abuse and specifically NAB cases, critical gaps in data collection will make it difficult to understand the scope of the problem and to develop effective policies.

To better detect NAB in children, we must implement standardized screening tools. The Netherlands successfully uses the SPUTOVAMO framework, which could also be implemented in Central Europe. SPUTOVAMO stands for Social history, Previous injuries, Unusual explanation, Timing, Object used, Verbal interactions, Age of the injury, Matching the story with the injury, and Other signs of abuse or neglect. SPUTOVAMO helps catch red flags such as delayed medical care, inconsistent explanations of the injury, and specific burn patterns, e.g., immersion scalds with sharp “waterline” edges, or “stocking” and “glove” patterns from forced immersion [10–12]. Within this framework, we can combine training programs for healthcare providers with multidisciplinary collaborations between social workers, pediatricians, and law enforcement to create a cohesive response system. Trainings, regular workshops or online courses should teach healthcare providers to recognize signs of NAB and other abuse, explain the rules for reporting suspected abuse and documenting cases, and demonstrate how healthcare providers can coordinate with social workers and police. Centralized databases that track abuse cases could also inform public health policies and decisions about how to allocate resources.

In Central Europe, public healthcare systems have different levels of resources, and offer and require different training, which means they could use SPUTOVAMO differently unless they receive standardized training. Each country also has different legal regulations for reporting abuse, so the tool must be adapted to fit local laws and processes. SPUTOVAMO must be adapted and translated into terms clear and familiar to the healthcare providers who will use it. When the tool is adapted, it must account for economic differences and unequal access to healthcare in the region that can influence how and when abuse is identified. Obstacles to implementing a standardized protocol for identifying NAB in Central Europe may include resistance to change among practitioners, limited funding for training and resources, and the stigma of child abuse. Strong governmental and institutional support for the standardization effort would move the project forward. The standardization effort must be funded, healthcare workers comprehensively trained, and collaboration between medical, social, and legal entities fostered to ensure standardized practices are universally adopted.

Though NAB in children is an urgent public health issue that needs immediate, coordinated action, lack of standardized screening protocols in Central Europe has made it difficult to identify and manage cases of abuse. Implementing standardized protocols will safeguard children and increase public confidence in the region’s ability to detect, treat, and protect children who suffer abuse by burning. Bu we must act now, since ever NAB case we miss places the wellbeing of our youngest and most vulnerable population at risk.

## Data Availability

The original contributions presented in the study are included in the article/supplementary material, further inquiries can be directed to the corresponding author.

## References

[1] American Burn Association. Scald Statistics and Data Resources (2018). Available online at: https://ameriburn.org/wp-content/uploads/2018/12/nbaw2019_statsdataresources_120618-1.pdf (Accessed July 8, 2025).

[2] Keen JH LendrumJ WB. Inflicted Burns and Scalds in Children. Br Med J (1975) 4:268–9. 10.1136/bmj.4.5991.268 1192018 PMC1675100

[3] Deitch EA Staats M. Child Abuse Through Burning. J Burn Care Rehabil (1982) 3:89–94. 10.1097/00004630-198203000-00005

[4] Hobson MI Evans J Stewart IP. An Audit of Non-Accidental Injury in Burned Children. Burns (1994) 20:442–5. 10.1016/0305-4179(94)90039-6 7999275

[5] Wibbenmeyer L Liao J Heard J Kealey L Kealey G Oral R. Factors Related to Child Maltreatment in Children Presenting with Burn Injuries. J Burn Care Res (2014) 35:374–81. 10.1097/BCR.0000000000000005 24823333

[6] Chester DL Jose RM Aldlyami E King H Moiemen NS. Non-Accidental Burns in Children—Are We Neglecting Neglect? Burns (2006) 32(2):222–8. 10.1016/j.burns.2005.08.018 16448766

[7] Allshouse M Rouse T Eichelberger R. Childhood Injury: A Current Perspective. Ped Emer Care (1993) 9:159–64. 10.1097/00006565-199306000-00012 8346091

[8] Hummel IIIRP Greenhalgh DG Barthel PP DeSerna CM Gottschlich MM James LE Outcome and socio-Economic Aspects of Suspected Child Abuse Scald Burns. J Burn Care Rehabil (1993) 14:121–6. 10.1097/00004630-199301000-00026 8454659

[9] Friedman SB Morse CW. Child Abuse: A Five-Year Follow-Up of Early Case Finding in the Emergency Department. Pediatrics (1974) 54:404–10. 10.1542/peds.54.4.404 4412414

[10] Bousema S Stas HG van de Merwe MH Oen IMMH Baartmans MGA van Baar ME Epidemiology and Screening of Intentional Burns in Children in a Dutch Burn Centre. Burns (2016) 42(6):1287–94. 10.1016/j.burns.2016.01.009 27211360

[11] Teeuw AH Kraan RBJ van Rijn RR Bossuyt PMM Heymans HSA. Screening for Child Abuse Using a Checklist and Physical Examinations in the Emergency Department Led to the Detection of More Cases. Acta Paediatr (2019) 108(2):300–13. 10.1111/apa.14495 29992712

[12] Jenny C. Cutaneous Manifestations of Child Abuse. In: Reese RM Ludwig S, editors. Child Abuse: Medical Diagnosis and Management. 2nd ed. Philadel- phia, PA: Lippincott Williams and Wilkins (2001). p. 38.

